# Impaired glymphatic function in the early stages of disease in a TDP-43 mouse model of amyotrophic lateral sclerosis

**DOI:** 10.1186/s40035-022-00291-4

**Published:** 2022-03-15

**Authors:** Akram Zamani, Adam K. Walker, Ben Rollo, Katie L. Ayers, Raysha Farah, Terence J. O’Brien, David K. Wright

**Affiliations:** 1 Department of Neuroscience, Central Clinical School, Monash University, Melbourne, VIC 3004 Australia; 2 Queensland Brain Institute, The University of Queensland, St Lucia, QLD 4072 Australia; 3 The Murdoch Children’s Research Institute, The Royal Children’s Hospital, Parkville, VIC 3052 Australia; 4 Department of Pediatrics, The University of Melbourne, Parkville, VIC 3052 Australia; 5 Department of Medicine, The Royal Melbourne Hospital, The University of Melbourne, Parkville, VIC 3052 Australia

**Keywords:** Magnetic resonance imaging, Diffusion-weighted imaging, Neurodegeneration, Cerebrospinal fluid, Telomere

## Abstract

**Background:**

Multiple lines of evidence suggest possible impairment of the glymphatic system in amyotrophic lateral sclerosis (ALS). To investigate this, we used in vivo magnetic resonance imaging (MRI) to assess glymphatic function early in the course of disease in a transgenic mouse with doxycycline (Dox)-controlled expression of cytoplasmic human TDP-43 (hTDP-43ΔNLS), mimicking the key pathology implicated in ALS.

**Methods:**

Adult TDP-43 transgenic and littermate monogenic control mice underwent longitudinal multimodal MRI one and three weeks after the cessation of Dox feed, together with weekly rotarod assessments of motor performance. Glymphatic function was assessed using dynamic contrast-enhanced MRI to track the clearance of an MR contrast agent injected into the cisterna magna.

**Results:**

Compared to their littermate controls, TDP-43 mice exhibited progressive neurodegeneration including that within the primary motor cortex, primary somatosensory cortex and corticospinal tract, significant weight loss including gastrocnemius atrophy, and shortened telomere length. Furthermore, in the presence of this ALS-like phenotype, these mice have significantly disrupted glymphatic function.

**Conclusions:**

Although the relationship between glymphatic clearance and ALS disease progression remains to be elucidated, these changes occurred very early in the disease course. This provides initial evidence to suggest that the glymphatic system might be a potential therapeutic target in the treatment of ALS.

## Background

Neurodegenerative diseases are chronic and inexorable conditions characterized by the presence of insoluble aggregates of abnormally ubiquitinated and phosphorylated proteins [1]. Transactive response DNA-binding protein 43 (TDP-43) is one such protein implicated in the neurodegenerative process and the presence of TDP-43 inclusions in neurons is a hallmark finding in amyotrophic lateral sclerosis (ALS) [2, 3]. Recent evidence also points to a prion-like self-propagation of TDP-43 misfolding, either by the circulatory system, cell-to-cell contact, or via the interstitial or cerebrospinal fluids (CSF) [1, 4–6]. As protein aggregation occurs before the onset of brain damage and motor symptoms, new therapeutic strategies targeting the spread of disease across the brain (including eliminating seed proteins and blocking cell-to-cell spread) are of vital importance.

One potential therapeutic target is the glymphatic system, a CSF-based waste clearance system for the brain [7]. Mediated by a unique system of astrocyte-specific aquaporin-4 water channels, the glymphatic system is largely dormant during wakefulness but highly active during sleep, working to clear waste byproducts from the brain through the flow of CSF [7]. Preclinical studies have shown that amyloid-β, a protein implicated in neurodegenerative diseases including Alzheimer’s disease, is cleared from the brain by the glymphatic system [7] and that in aged mice, amyloid-β clearance is dramatically slowed [8]. As the prevalence of ALS also increases with age, sleep disturbances are exceedingly common in ALS [9] and given that a single night of sleep deprivation can result in amyloid-β accumulation linked with Alzheimer’s disease [10], we hypothesize that glymphatic clearance is also impaired in ALS.

To investigate this, we assessed glymphatic function in a TDP-43 transgenic mouse model of ALS. Specifically, these mice have doxycycline (Dox)-suppressible neurofilament heavy chain (*NEFH*) promoter-driven expression of human TDP-43 (hTDP-43) harboring a defective nuclear localization signal (hTDP-43ΔNLS). When these mice are removed from Dox feed, hTDP-43 becomes expressed, resulting in the accumulation of insoluble phosphorylated cytoplasmic TDP-43 in neurons of the brain and spinal cord and loss of endogenous nuclear mouse TDP-43 [11]. Continued hTDP-43 expression has been shown to result in much of the pathophysiology seen in ALS, including brain atrophy, cortical and spinal motor neuron loss, muscle denervation, and progressive motor impairments, leading to premature death.

We performed longitudinal multimodal magnetic resonance imaging (MRI) one and three weeks after the cessation of Dox feed, together with weekly rotarod assessments of motor performance. Glymphatic function was assessed using dynamic contrast-enhanced MRI (DCE-MRI) to track the clearance of an MR contrast agent injected into the cisterna magna [12]. DCE-MRI was performed following the second multimodal imaging session three weeks after the cessation of Dox feed.

## Methods

### Animals

This study included 7 NEFH + /NLS + (rNLS8) double transgenic mice with Dox-suppressible expression of hTDP-43ΔNLS (referred to as ‘TDP-43 mice’ throughout) and 5 NEFH-/NLS + single transgenic littermate controls (Jackson Laboratories, *NEFH*-tTA line 8, stock #025,397 and *tetO*-TARDBP* line 4, stock #014,650), on a pure C57Bl/6JAusb background following ~ 10 generations of backcrossing. The phenotypes of these mice were consistent with that described in detail previously [11]. Mice were all males, aged 10–12 weeks and group-housed under a 12 h/12 h light/dark cycle with ad libitum access to food and water. All of the 12 mice were provided with 200 mg/kg Dox-containing chow (Diet SF11-059, Specialty Feeds, Glen Forrest, Western Australia, Australia) until the start of their experimental paradigm when they were switched to standard chow.

### Experimental design

Mice were placed on standard chow for three weeks, by which time the TDP-43 mice are expected to demonstrate decreased body mass, hindlimb clasping and sensorimotor deficits [11]. The mice underwent weekly behavioral testing and in vivo MRI at 1 and 3 weeks after the cessation of Dox feed. DCE-MRI was performed following the second MRI at three weeks after the cessation of Dox feed, to assess glymphatic function. At the end of the DCE-MRI imaging session, the mice were deeply anaesthetized with an intraperitoneal injection of sodium pentobarbital (Lethabarb, Virbac, Australia). A sample of ear tissue was collected and stored at – 80 °C. Gastrocnemius muscles and spleens were collected and weighed.

### Behavioral testing

An accelerating rotarod test was performed to assess sensorimotor coordination, balance and stamina. Mice were placed on a rotating cylinder with 3-cm diameter (Rotarod-RS; Panlab, Harvard Bioscience, Holliston, MA) that accelerated by 1 rpm every 8 s from an initial speed of 5 rpm. The cylinder continued to accelerate until the mouse fell off. The test was repeated three times at 10-min intervals. The latency to fall was recorded for each test and the average of all three tests was reported. Following the rotarod test, the mice were assessed for hindlimb clasping as described previously [11]. Behavioral assessments were performed during the light cycle at 1, 2 and 3 weeks after cessation of Dox feed.

### MRI

In vivo imaging was performed with a 9.4 T Bruker MRI, at 1 and 3 weeks after cessation of Dox feed, and included T_2_^*^-weighted and diffusion-weighted imaging (DWI) acquisitions. Mice were anesthetized using isoflurane in a carrier gas of 100% oxygen with body temperature maintained using a hot water system. Depth of anesthesia was monitored by measuring the respiration rate with a pressure-sensitive pillow positioned under the diaphragm (SA Instruments Inc., Stony Brook, NY). 3D T_2_^*^-weighted images were acquired in the axial plane with a multi-gradient echo sequence and parameters: repetition time (TR) = 70 ms; echo time (TE) = 2 ms; echo spacing = 3 ms; 10 echoes; field of view (FOV) = 16 × 16 × 8 mm^3^; and isotropic resolution = 125 × 125 × 125 µm^3^. DWI was acquired in ~ 10 min using a single-shot 2D diffusion tensor imaging (DTI)-echo planar imaging sequence. Two b_0_ images and two diffusion shells (b-value = 1500 s/mm^2^ and 3000 s/mm^2^) were acquired with 81 directions, δ = 3.5 ms and Δ = 12 ms. Other imaging parameters were adjusted to give an isotropic resolution of 250 µm^3^ and included: TR = 3600 ms; TE = 25 ms; and FOV = 16 × 16 mm^2^.

At 3 weeks post-Dox, in vivo DCE-MRI was also performed. A baseline DCE-MRI scan was acquired over the whole-brain using a 3D FLASH sequence with the following imaging parameters: TR = 12 ms; TE = 2.1 ms; flip angle = 15°; FOV = 19.2 × 19.2 × 14.4 mm^3^; matrix = 128 × 128 × 96; resolution = 150 × 150 × 150 µm^3^; and number of excitations = 2.

The mouse was then withdrawn from the magnet and the cisterna magna cannulated using a 30G needle, connected to polyethylene tubing (BPTE-10, Instech Laboratories Inc., Plymouth Meeting, PA) filled with Magnevist (gadopentetate dimeglumine; Bayer AG, Berlin, Germany) [33]. Ten microlitres of Magnevist (20 mM) was infused over 10 min using a perfusion pump and a Hamilton syringe. The needle was left in place for 5 min after infusion, then slowly withdrawn. The mouse was repositioned inside the MRI scanner and DCE-MRI imaging resumed 30 min after the start of the infusion, with T_1_-weighted images acquired every 5 min for 170 min. Isoflurane anesthesia was maintained throughout the imaging process.

### MRI analysis

The T_2_^*^-weighted images were registered to the Waxholm Space adult C57BL/6 J mouse brain atlas using symmetric diffeomorphic normalization. The inverse diffeomorphisms were then used to register the atlas labels to native space. Volumes were calculated for four regions of interest (ROIs), including the whole brain, neocortex, hippocampus and hypothalamus. Additionally, whole-brain tensor-based morphometry analysis was performed on smoothed log-Jacobian images calculated from the diffeomorphisms as described previously [13].

DWI processing was performed with the MRtrix3 [14] and FSL packages and included denoising, Gibbs correction and eddy current correction followed by a fully automated estimation of three tissue response functions. Fibre orientation distributions were estimated using multi-tissue constrained spherical deconvolution and normalized, and a study-specific template was generated for statistical analysis of the fixel-based metric fibre density and cross-section (FDC) [15]. To assess any differences in microstructural changes in TDP-43 and control mice over time, the week-3 fixel values were subtracted from week-1 fixel values, and statistical testing performed on the delta values (ΔFDC).

An a priori analysis of the corticospinal tracts was also performed. Left and right corticospinal tract streamlines were segmented as described previously [13]. The segmented streamlines were used to identify the corticospinal tract fixels, i.e. those fixels aligned parallel to the streamlines of interest—and the maximum FDC value was calculated for each voxel. FDC values were then sampled along the corticospinal tracts at 20 equidistant sections from the pons to the motor cortex.

To correct for movement over time, DCE-MRI T_1_-weighted images were aligned using a rigid transform. A mapping between the mean T_1_-weighted image and the Waxholm Space adult C57BL/6 J mouse brain atlas, down sampled to 43 × 43 × 43 µm^3^, was generated using symmetric image normalization and the atlas segmentations transformed into T_1_-weighted image space. The average signal increase over baseline was calculated at each time point using MATLAB for 6 atlas-defined ROIs: the whole brain, excluding the ventricles; pons; amygdala; olfactory bulb; thalamus; and cerebellum.

## Telomere analysis

DNA extraction from 25-mg ear tissue was performed using DNeasy® Blood and Tissue kit (QIAGEN, Hilden, Germany) according to the manufacturer’s instructions. The quality and quantity of DNA was measured using the QIAxpert system and samples with an A260/A280 ratio of 1.7–2 were used. DNA was diluted to 20 ng/µl in Tris–EDTA buffer solution for further analysis. Two master mixes containing telomere repeat copy and a single copy gene (36B4) were used. The qPCR mix contained 1 µl DNA, 1× SYBR Green master mix (Promega, Madison, WI), and forward and reverse primers. The final primer concentrations were as follows: telomere forward 270 nM, telomere reverse 900 nM, 36B4 forward 300 nM and 36B4 reverse 500 nM.

Auto-aliquoting was performed using the QIAgility liquid handling robot and samples were run in duplicates with primer sequences as described previously [16]. The thermocycling conditions for telomere were 1 cycle at 95 °C for 3 min, 30 cycles of 95 °C for 15 s and 54.8 °C for 1 min followed by the melting curve, and for 36B4 were 1 cycle at 95 °C for 3 min, 30 cycles of 95 °C for 15 s and 58.4 °C for 1 min followed by the melting curve. Telomere length was determined by comparing telomere to 36B4 as described previously [17].

### Statistical analyses

Voxel-wise analysis of tensor-based morphometry was performed using the FSL’s Randomise with threshold-free cluster enhancement [18], and results were fully corrected for multiple comparisons. Changes in ΔFDC were assessed using connectivity-based fixel enhancement with results fully corrected for family-wise error (FWE). Corticospinal tract analyses of FDC were tested using a general linear model with time as repeated measure (1 and 3 weeks post-Dox) and genotype (TDP-43, control) and position along the tracts (p1 to p20) as factors. Statistical testing was performed using the IBM SPSS Statistics for Macintosh, Version 27.0.

Two-way ANOVAs or mixed-effects analyses with genotype (TDP-43 *vs* control) as the between-subject factor and time (1 week vs 3 weeks post-Dox) as the within-subject factor were used to assess behavior and ROI-based measures. Šídák’s multiple comparison tests were performed where necessary. Data on telomere length were analyzed with an unpaired *t*-test. Statistical testing for these comparisons was performed using GraphPad Prism version 9.1.0 software (GraphPad, San Diego, CA). Significance for all tests was set at *P* < 0.05 and all results are presented as mean ± standard error of the mean (SEM).

## Results

### TDP-43 mice exhibited ALS-like phenotype with symptoms deteriorating over time

**Fig. 1:**
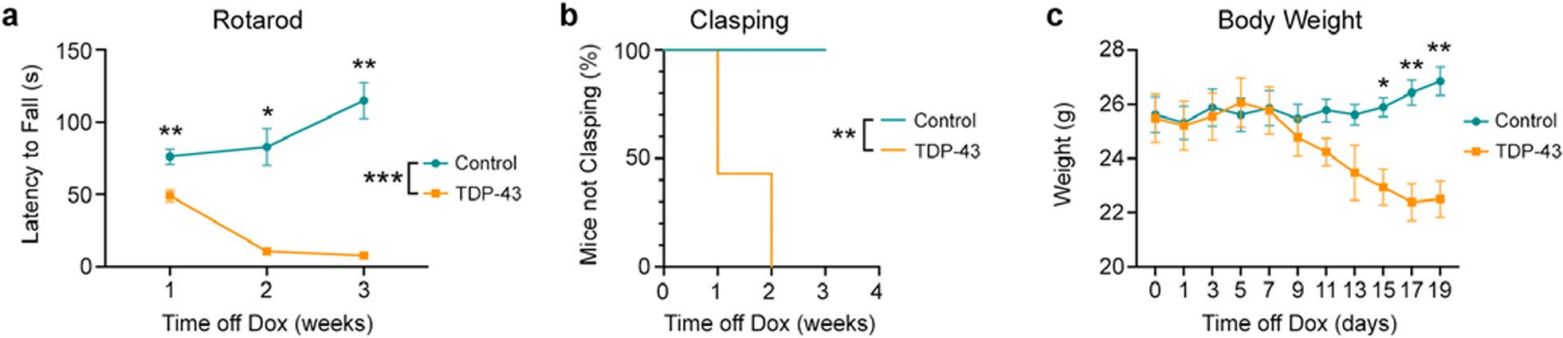
Motor performance is impaired in TDP-43 mice. **a** TDP-43 mice (*n* = 7) performed significantly worse in the rotarod task than their littermate controls (*n* = 5), which improved over time. Two-way ANOVA revealed a significant interaction between time and genotype (*P* < 0.0001), and a main effect of genotype with control mice running for significantly longer than TDP-43 mice (*P* < 0.0001). **b** Survival plot showing the percentage of mice not clasping at 1, 2 and 3 weeks post-Dox cessation. **c** The body weight of mice measured on the day of, and every two days after, the cessation of Dox feed. Two-way ANOVA demonstrated a significant interaction between genotype and time (*P* < 0.0001). Main effects of genotype are indicated by lines with asterisk between genotype symbols in figure legends. Significant results following Šídák’s multiple comparison testing are indicated by asterisk(s) at corresponding time points. *P < 0.05, **P < 0.01, ***P < 0.001

To determine if TDP-43 mice develop a progressive motor phenotype, we assessed motor coordination, balance and stamina using the rotarod test at 1, 2 and 3 weeks after the cessation of Dox feed. Two-way ANOVA revealed a significant interaction between time and genotype (*F*_2,20_ = 27.05, *P* < 0.0001), with control mice running for significantly longer than TDP-43 mice (*F*_1,10_ = 99.74, *P* < 0.0001) and improving over time (Fig. 1a). Šídák’s multiple comparisons test revealed that the TDP-43 mice performed significantly different from control mice at all time points after cessation of the Dox feed.

Hindlimb clasping is a well-established marker for neurodegenerative disease progression [2, 11, 19] and we found that the TDP-43 mice also developed early-onset hindlimb clasping over time (Fig. 1b). While none of the control mice presented with hindlimb clasping during the study, 57% of the TDP-43 mice demonstrated hindlimb clasping at 1 week post-Dox feed, with the remainder being symptomatic at 2 weeks post-Dox. The TDP-43 mice also demonstrated significant body weight loss compared to the control mice (Fig. 1c). Two-way ANOVA revealed a significant interaction between time and mouse genotype (*F*_2,20_ = 14.66, *P* < 0.0001), with the TDP-43 mice having significantly lower body weight beginning from 15 days after cessation of the Dox feed.

### Structural imaging revealed progressive atrophy of the grey matter in TDP-43 mice

**Fig. 2:**
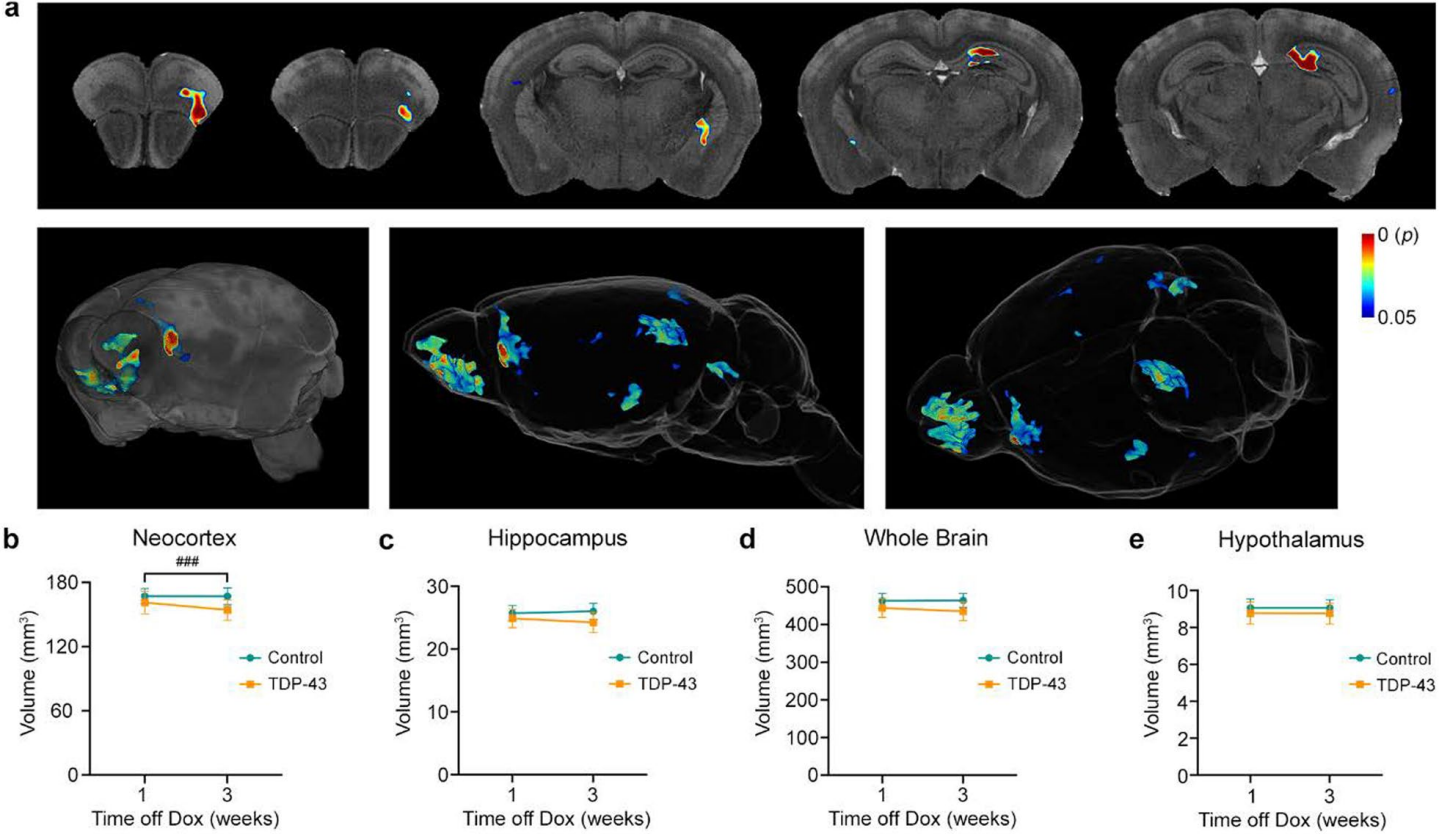
TDP-43 mice exhibited progressive brain atrophy. **a** Tensor-based morphometry analysis revealed foci of significant atrophy in TDP-43 mice (*n* = 7) compared to littermate controls (*n* = 5) at 3 weeks after the cessation of Dox feed. Results are shown overlaid on the Waxholm Space adult C57BL/6 J mouse brain atlas (top row), a 3D volume render (left, bottom) and glass brain (middle and right, bottom). The color bar shows FWE-corrected *P*-value. **b–e** Region-of-interest analysis demonstrated significant interactions between group and time in the neocortex (*P* = 0.0011) and hippocampus (*P* = 0.0207). No significant differences were observed for the whole brain or hypothalamus. ^###^*P* < 0.001, main effect of time off Dox

To understand the timing and pattern of possible morphological brain changes in TDP-43 mice, we analyzed structural images using tensor-based morphometry at 1 and 3 weeks after cessation of the Dox feed. This exploratory method revealed foci of significant atrophy in TDP-43 mice compared to control mice at the latter time point (Fig. 2a). The affected regions included, from anterior to posterior, the olfactory bulb, frontal association cortex, lateral and dorsolateral orbital cortex, agranular insular cortex, globus pallidus, hippocampus, dorsal subiculum, secondary visual cortex and cerebellum. No foci of hypertrophy were detected. No atrophy or hypertrophy was detected at 1 week after cessation of the Dox feed. Interestingly, no foci of atrophy were detected in the primary motor cortex.

In addition to the exploratory analysis, we also performed a volumetric analysis of atlas-defined ROIs. Regions were selected a priori and included the neocortex, hypothesized to be the structure most likely to be affected early in the disease course [20], the hippocampus, the whole-brain, and the hypothalamus which contains the majority of orexin-producing neurons involved in wakefulness [21]. Two-way ANOVA revealed significant genotype and time interactions for volumes of both the neocortex (*F*_1,10_ = 20.29, *P* = 0.0011; Fig. 2b) and hippocampus (*F*_1,10_ = 7.53, *P* = 0.0207; Fig. 2c). No differences were observed between TDP-43 and control mice at 1 or 3 weeks post-Dox feed using Šídák’s multiple comparison testing. No significant differences were found for the whole brain (Fig. 2d) or the hypothalamus (Fig. 2e).

### In vivo DWI demonstrated microstructural white matter changes in the brains of TDP-43 mice

DWI methods can detect microstructural changes to the white matter in the absence of any macroscopic changes on conventional structural images [22, 23]. Using multi-tissue constrained spherical deconvolution, we estimated a fibre orientation distribution for each voxel. Unlike DTI-derived metrics, which contain contributions from multiple fibre bundles in each voxel (often orientated in different directions), constrained spherical deconvolution allows diffusion metrics to be extracted for each *fixel* (i.e. individual fibre bundles within each voxel). Further, these metrics can be tested using fixel-based analysis to localize changes to individual fibre bundles and are therefore less likely to be confounded by crossing fibre bundles.

**Fig. 3:**
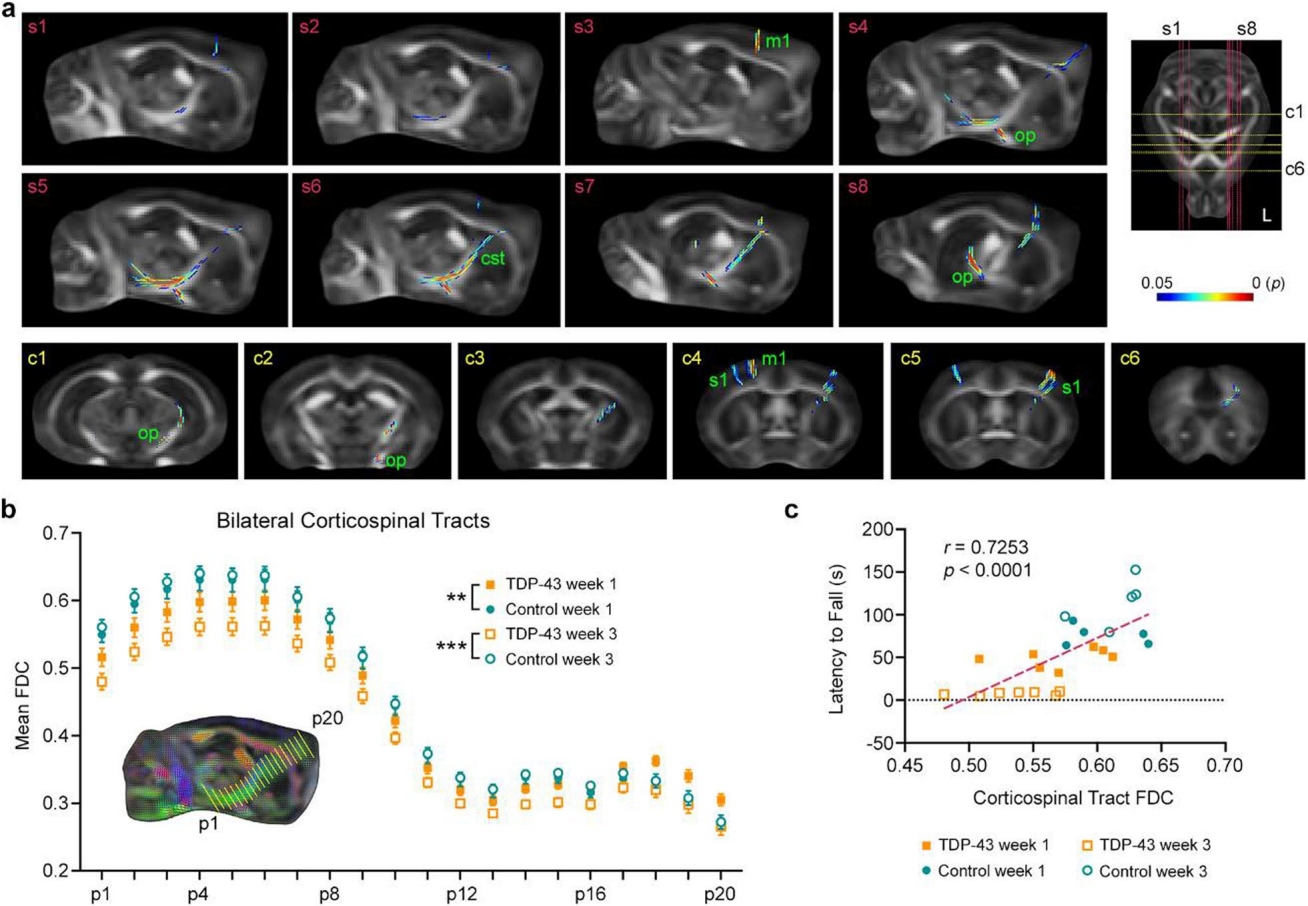
DWI reveals progressive microstructural neurodegeneration in TDP-43 mice. **a** Using connectivity-based fixel enhancement, we compared the change in fibre density and cross-section (FDC) from 1 to 3 weeks after the cessation of Dox feed. When compared to their littermate controls (*n* = 5), the TDP-43 mice (*n* = 7) demonstrated significantly reduced FDC in the left corticospinal tract (cst), left optic pathway (op), primary motor cortex (m1) and primary somatosensory cortex (s1). Significant fixels are shown overlaid on the template fractional anisotropy image. The position of sagittal sections (s1–s8, red lines) and coronal sections (c1–c6, yellow lines) are indicated on the axial section at right (‘L’ = left side of the brain). The color bar shows FWE-corrected *P*-value. **b** Mean FDC values plotted for each position (from p1 to p20) along the bilateral corticospinal tracts demonstrating progressive damage (i.e., reduced FDC values) to the corticospinal tracts of TDP-43 mice over time. **P < 0.01, ***P < 0.001, FDC values of TDP-43 mice significantly different from control mice. **c** The caudal corticospinal tract integrity, calculated as the mean FDC value from position p1 to p5, also correlated with motor performance as measured on the rotarod. *n* = 7 TDP mice, *n* = 5 control mice

We compared the change in white matter connectivity (i.e., ΔFDC) between TDP-43 mice and control mice using connectivity-based fixel enhancement (Fig. 3). Results showed significantly greater ΔFDC in TDP-43 mice over time. The affected fibre bundles included the left corticospinal tract (Fig. 3a, panels s4–s7) and left optic pathway (Fig. 3a, panels s4 to s8, and c1 and c2). The left and right cortices were also affected, including fibres in the primary motor cortex and primary somatosensory cortex (hindlimb and forelimb regions, Fig. 3a, panels c4 and c5), with TDP-43 mice demonstrating significantly greater ΔFDC between 1 and 3 weeks post-Dox.

As corticospinal tract degeneration is a feature of ALS [13, 24, 25], we also performed an a priori analysis of these white matter fibre bundles. Corticospinal tract fixels demonstrated a progressive reduction in FDC values in TDP-43 mice compared to controls. We measured FDC values at 20 equidistant points (p1, p2, …, p20) along the left and right corticospinal tracts. For each point, we then calculated the average FDC value from both hemispheres. There was a significant interaction between genotype and time (*F*_1,200_ = 202.655, *P* < 0.001, Wilks’ Λ = 0.497), with the TDP-43 mice having significantly decreased FDC at both 1 and 3 weeks post-Dox (*P* = 0.001 and *P* < 0.001, respectively). Although there was no interaction for either time × position or time × position × genotype, visual inspection of the mean FDC values showed that the FDC decrease occurred at the caudal end of the corticospinal tract (Fig. 3b).

Finally, we investigated whether the observed corticospinal tract degeneration (i.e., average FDC values along the caudal section) correlated with the deteriorating motor deficits (i.e., latency to fall). Results showed that the mean FDC values correlated with the latency to fall (*r*_22_ = 0.7253, *P* < 0.0001) and this correlation also held true within the TDP-43 mice only (*r*_12_ = 0.5561, *P* = 0.0389) (Fig. 3c).

### DCE-MRI revealed altered glymphatic clearance in symptomatic TDP-43 mice

**Fig. 4:**
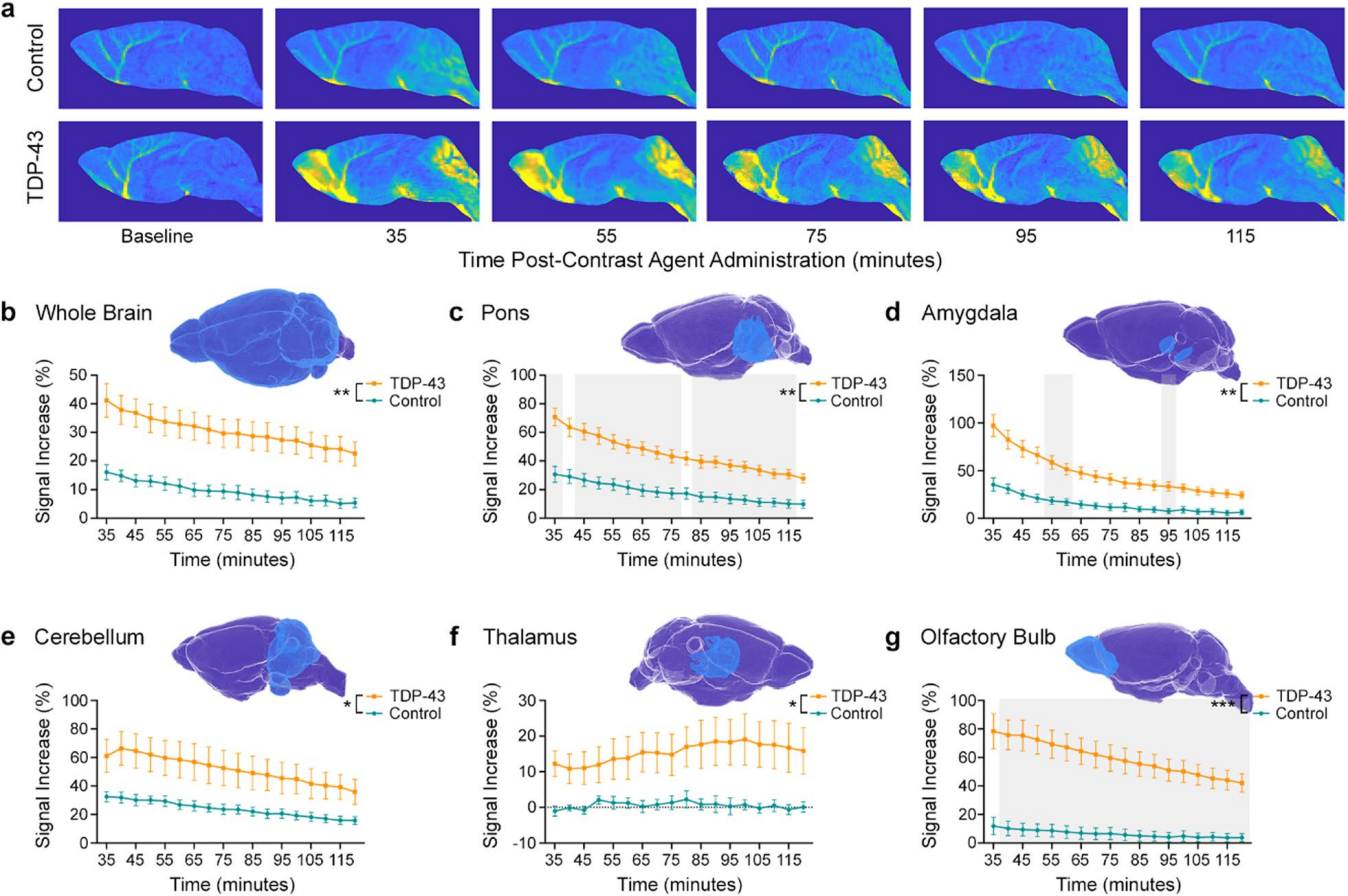
Dynamic contrast-enhanced MRI (DCE-MRI) revealed impaired glymphatic clearance in TDP-43 mice. **a** Representative DCE-MRI images of control (top row) and TDP-43 (bottom row) mice over time after contrast agent administration. **b–g** Percent signal increase from baseline in the whole brain, pons, amygdala, cerebellum, thalamus and olfactory bulb. In all cases, mixed-effect analysis revealed a significant interaction between genotype and post-infusion time and main effects of both genotype (*P < 0.05, **P < 0.01, ***P < 0.001) and time. Each atlas-defined ROI is shown in blue above the corresponding graph. Grey backgrounds in panels **c**, **d**, and **g** indicate times for which multiple comparison testing demonstrated significant differences between TDP-43 mice (*n* = 5) and their littermate controls (*n* = 5)

Following in vivo MRI assessments of brain morphometry and white matter degeneration, we assessed glymphatic clearance using DCE-MRI. Cisterna magna cannulation, infusion of MR contrast agent and DCE-MRI were successfully performed in five TDP-43 mice and five control mice. Two mice were removed due to experimental error that resulted in no contrast agent administration. T_1_-weighted images demonstrated clear differences between the two groups (Fig. 4a). A delay in acquiring images in one TDP-43 mouse resulted in a single missing datapoint, and therefore statistical testing was performed using a mixed-effect analysis.

The signal increase from baseline, expressed as a percentage, was analyzed for six ROIs. In all cases, statistical testing revealed a main effect of post-infusion time, with *P* < 0.0001 for each. Over the whole brain (excluding the ventricles), a significant genotype and time interaction (*F*_17,135_ = 3.38, *P* < 0.0001) and a significant effect of genotype (*F*_1,8_ = 16.39, *P* = 0.0037) were observed (Fig. 4b). Ventral brain regions including the pons (Fig. 4c) and amygdala (Fig. 4d) exhibited a similar response over time, with significantly increased signal intensity in TDP-43 mice compared to their littermate controls. Mixed-effect analyses demonstrated significant genotype and time interactions (*F*_17,135_ = 5.83, *P* < 0.0001 and *F*_17,135_ = 15.52, *P* < 0.0001, respectively) as well as significant effects of genotype (*F*_1,8_ = 22.95, *P* = 0.0014 and *F*_1,8_ = 23.00, *P* = 0.0014, respectively). In the cerebellum (Fig. 4e), a significant interaction between genotype and post-infusion time was also recorded (*F*_17,135_ = 5.75, *P* < 0.0001), with a main effect of genotype (*F*_1,8_ = 5.84, *P* < 0.0420).

The percent signal change from baseline in the thalamus was significantly different between TDP-43 and control mice (Fig. 4f). In contrast to the control mice that showed almost no increase from baseline, the TDP-43 mice showed gradual increase in signal, peaking at ~ 100 min after contrast agent administration. Mixed-effect analysis revealed a significant interaction between genotype and post-infusion time (*F*_17,135_ = 3.21, *P* < 0.0001) and a significant main effect of genotype (*F*_1,8_ = 5.98, *P* < 0.0402). Finally, the olfactory bulb (Fig. 4g) also demonstrated a significant interaction between genotype and post-infusion time (*F*_17,135_ = 22.98, *P* < 0.0001), and a main effect of genotype (*F*_1,8_ = 31.08, *P* < 0.0005).

### Post-mortem investigations revealed reduced muscle mass and shorter telomere length in TDP-43 mice

**Fig. 5:**
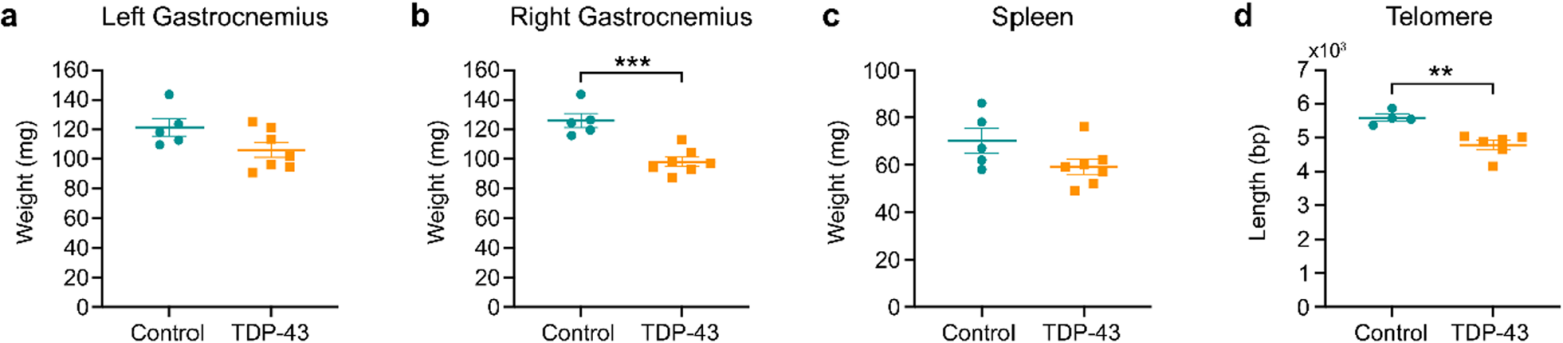
Post-mortem analyses revealed muscle atrophy and reduced telomere length in TDP-43 mice. **a**, **b** Although not reaching significance on the left-side, the right-side gastrocnemius muscles were significantly lighter in TDP-43 mice (*n* = 7) when compared to control mice (*n* = 5) at 3 weeks after the cessation of Dox feed. **c** TDP-43 mice also tended to have lighter spleens. **d** Telomere length was assessed using quantitative PCR on DNA extracted from ear tissue samples and found to be significantly shorter in TDP-43 mice (*n* = 6) when compared to control mice (*n* = 4). **P < 0.01, ***P < 0.001

Following DCE-MRI, the mice were sacrificed, and the gastrocnemius muscles (TDP *n* = 7, control *n* = 5) and spleens were collected and weighed (Fig. 5a–c). The right gastrocnemius muscles from the TDP-43 mice were significantly lighter than those from littermate controls (*t*_10_ = 5.10, *P* = 0.0005). TDP-43 mice also had a trend (*t*_10_ = 1.88, *P* = 0.0903) for smaller spleens than their littermate controls (Fig. 5c). The spleen weight (mean ± SEM) was 70.2 ± 5.18 mg for control mice and 59.29 ± 3.28 mg for TDP-43 mice.

As conjecture remains surrounding how telomeres are affected in ALS, we also assessed telomere length using quantitative PCR on DNA extracted from ear tissue samples (Fig. 5d). Unpaired *t*-test analysis revealed that the TDP-43 mice (*n* = 6) had significantly shorter telomeres (*t*_8_ = 4.26, *P* = 0.0028) than their littermate controls (*n* = 4). The telomere length (mean ± SEM) was 5593 ± 103.5 bp for control mice and 4784 ± 137.3 bp for the TDP-43 mice.

## Discussion

In this study, we assessed glymphatic function in a transgenic mouse model with Dox-suppressible expression of hTDP-43. The mice were removed from Dox feed to permit hTDP-43 expression, then they were assessed longitudinally using rotarod testing and underwent in vivo multimodal MRI followed by DCE-MRI at 3 weeks post-Dox cessation. Our results demonstrate that the TDP-43 mice exhibited significantly altered glymphatic function, progressive neurodegeneration, deteriorating motor symptoms, significant weight loss, and shortened telomere length when compared to their littermate controls, very early in the disease course.

Waste protein inclusions are a hallmark of all neurodegenerative diseases and recent efforts have recognized the importance of the glymphatic system for protein regulation and brain clearance. This brain-wide network of perivascular space facilitates the exchange of CSF and interstitial fluid, clearing waste from the CSF while also assisting in distributing compounds critical to neurological function. Here, we used DCE-MRI to investigate glymphatic function following the injection of an MR contrast agent, Magnevist, into the cisterna magna, a technique first demonstrated by Iliff and colleagues [12].

As hypothesized, glymphatic function was found to be altered in TDP-43 mice at 3 weeks post-Dox cessation, a time point prior to overt neurodegeneration (4 weeks) and very early in the disease course in this model [11]. Although this is the first in vivo study of glymphatic function in ALS and one of the few DCE-MRI studies conducted in mice, similar results demonstrating increased signal with impaired glymphatic function have been observed in rodents previously [26, 27]. One recent study employed DCE-MRI to investigate the effect of the circadian light/dark cycle in awake rats [28]. This study also showed similar brain-wide kinetics, with increased signal during the dark (awake) phase—when glymphatic clearance is known to be reduced. Although we did not acquire images during contrast agent infusion and hence were unable to similarly quantify the time to peak signal increase, we demonstrated significant interactions between time and genotype for each of the regions analyzed, and further, found a delay in contrast agent uptake in the thalamus, suggesting impaired glymphatic function in TDP-43 mice.

Our preliminary results highlight the need for additional experiments, at multiple time points, to improve our understanding of the relationship between ALS and glymphatic system function, and how this relationship evolves with disease progression. In addition to measuring signal change during contrast agent infusion for the spatial quantification of measures such as peak signal increase and the time to peak signal increase, future experiments should also consider the use of α_2_ agonists such as xylazine and dexmedetomidine [29, 30]. When compared to isoflurane alone, dexmedetomidine and low-dose isoflurane significantly increased glymphatic transport [30] and the interstitial fluid space [31] and hence, may better mimic sleep [32].

In addition to DCE-MRI, we performed in vivo MRI at 1 and 3 weeks after the cessation of Dox feed. Structural analysis of hippocampal volume demonstrated an interaction between genotype and time, with volumes increasing in control mice and decreasing over time in TDP-43 mice. Further, tensor-based morphometry also revealed atrophy of the hippocampus at 3 weeks post-Dox cessation. Altogether, these results suggest a possible loss of hippocampus neurons in TDP-43 mice over time. Hippocampal pathology is well documented in ALS patients primarily at later stages of disease [3, 20, 33–35] and a recent experimental study also demonstrated significant neuronal loss and atrophy of the hippocampus, after intracranial injection of recombinant adeno-associated virus serotype 9 containing human wild-type TDP-43, into the hippocampus of *CAMKII*-tTa transgenic mice [36]. Expression of a C-terminal fragment of TDP-43 found in the brains of frontotemporal lobar degeneration cases, has also been shown to cause a specific loss of hippocampal dentate gyrus neurons in mice, although the relevance for this finding to ALS remains unclear [37].

DWI has demonstrated potential as a sensitive biomarker for neurodegeneration, identifying microstructural changes to the white matter in the absence of any macroscopic changes on conventional radiological images [22, 23]. We assessed the fixel-derived metric FDC using both exploratory whole-brain and a priori analyses. As the name suggests, changes in the combined fibre density and cross-section measure reflect both a macroscopic change to the white matter bundle and a microstructural change due to increased or decreased axon fibre population [15, 38]. Further, as a fixel-derived metric, FDC can be assessed for specific fibre bundle orientations and hence, specific white matter tracts of interest.

The corticospinal tract is the major neuronal pathway controlling movement and has previously been implicated in DWI studies of ALS [13, 24, 25, 39]. Using connectivity-based fixel analysis, a whole-brain exploratory method, we found significantly reduced FDC in the left corticospinal tract of TDP-43 mice over time. Additionally, hypothesis-driven tract-of-interest analyses of bilateral corticospinal tract FDC values also demonstrated significant reductions in TDP-43 mice compared to littermate controls. Post-hoc comparisons demonstrated that the TDP-43 mice had significantly reduced FDC in the corticospinal tracts at 1 week post-Dox cessation, largely at the caudal end, near the cerebral peduncles. These changes were observed in the absence of any structural change to the primary motor cortices on T_2_^*^-weighted imaging, consistent with the hypothesis that axonopathy may precede the degeneration of neuronal cell bodies—i.e. ‘dying-back pathology’ [19, 40–42].

Consistent with the progressive corticospinal tract degeneration and the ALS phenotype, the TDP-43 mice also demonstrated progressive weight loss and deteriorating motor deficits when compared to controls. The motor performance of TDP-43 mice was significantly impaired compared to controls at 1 week post-Dox cessation, with over half also demonstrating a hind-limb clasping phenotype. At 3 weeks post-Dox cessation, clasping was evident in all TDP-43 mice and motor performance had further declined. Interestingly, the rotarod latency to fall correlated significantly with the mean caudal corticospinal tract FDC values, suggesting that the decline of motor function is closely associated with corticospinal tract degeneration.

As expected, the gastrocnemii weight of TDP-43 mice was significantly lighter than that of littermate controls. Post-mortem analysis also demonstrated a trend of smaller spleens in these mice, consistent with observations of reduced spleen weight late in the disease course in SOD1^G93A^ mice [43, 44]. Peripheral immune dysfunction is a pathogenic feature of clinical ALS, with altered levels of T-lymphocytes, monocytes and cytokines in the blood [45], and it is possible that similar immune system changes may occur in this TDP-43 mouse model.

Chronic systemic inflammation and immune cell exhaustion are also hypothesized sources of telomere shortening [46]. Telomeres are special chromatin structures formed at the ends of chromosomes, protecting them from degradation and recombination [47]. Each time a cell divides, the telomeres shorten, and hence telomere length is considered a measure of ageing [47]. Telomere shortening has been reported in other neurodegenerative diseases, including traumatic brain injury [17, 48, 49] and Alzheimer’s disease [50, 51]. Consistent with this, we found that telomere length was significantly shorter in TDP-43 mice when compared to controls.

In both sporadic ALS patients and healthy controls, telomere length has been shown to correlate with decreased telomerase reverse transcriptase expression [52]. In addition to maintaining telomere length, telomerase may also protect against cell damage. Increasing telomerase expression has been shown to delay disease onset in SOD1^G93A^ mice [53], while conversely, telomerase deletion accelerates disease progression in SOD1^G93A^ mice [54]. These results, together with our observation of reduced telomere length in TDP-43 mice, suggest telomere/telomerase dysfunction in the pathogenesis of ALS.

## Conclusions

In conclusion, we demonstrate that when compared to their littermate controls, the TDP-43 mice exhibit progressive neurodegeneration including that within the corticospinal tract that correlates with deteriorating motor symptoms, significant weight loss including muscle atrophy, and shortened telomere length—outcomes consistent with an ALS-like phenotype. Furthermore, we have shown that in the presence of this phenotype, these mice have significantly disrupted glymphatic function. While the relationship between glymphatic clearance and ALS disease progression remains to be elucidated, given that these changes occur very early in the disease course in the TDP-43 mice, these results provide initial evidence to suggest that, like other neurodegenerative disorders, the glymphatic system could be a potential therapeutic target in the treatment of ALS.

## Data Availability

The datasets used and/or analysed during the current study are available from the corresponding author on reasonable request.

## Abbreviations

ALS: Amyotrophic lateral sclerosis
CSF: Cerebrospinal fluid
DCE-MRI: Dynamic contrast-enhanced magnetic resonance imaging
Dox: Doxycycline
DWI: Diffusion-weighted imaging
FDC: Fibre density and cross-section
FWE: Family-wise error
MRI: Magnetic resonance imaging
ROI: Region of interest
TDP-43: Transactive response DNA-binding protein 43
NEFH: Neurofilament heavy chain
TE: Echo time
TR: Repetition time
SEM: Standard error of the mean
